# Supplementary figures and images for: Quercetin alleviates PM2.5-induced chronic lung injury in mice by targeting ferroptosis
Source: PeerJ. 2024 Jan 2;12:e16703. doi: 10.7717/peerj.16703 (PMC10768656; doi:10.7717/peerj.16703)

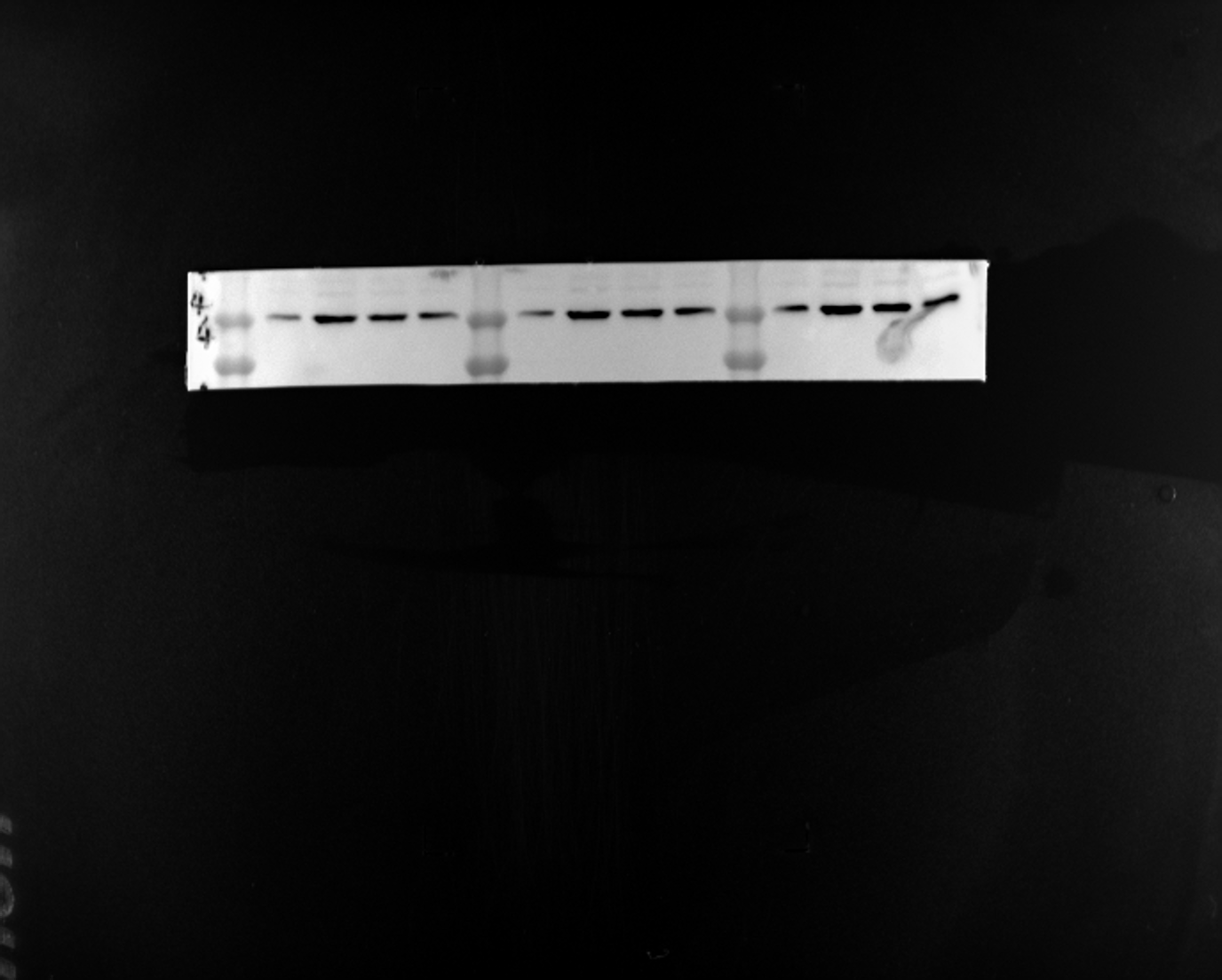

Supplement: Supplemental Information 2 [file peerj-12-16703-s002.zip › Figure 2-WB images/ACSL4/ACSL4.Tif]

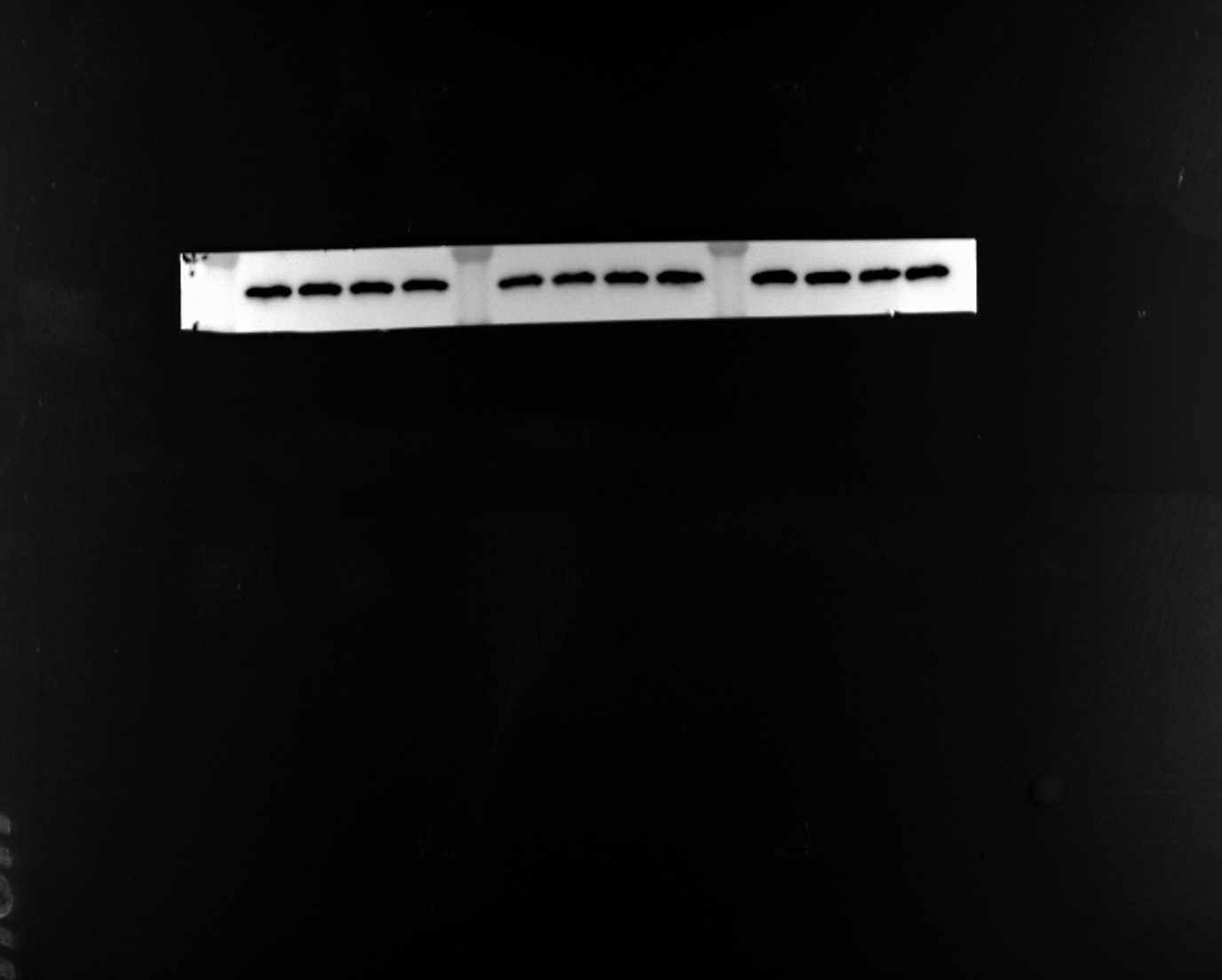

Supplement: Supplemental Information 2 [file peerj-12-16703-s002.zip › Figure 2-WB images/GAPDH/GAPDH.Tif]

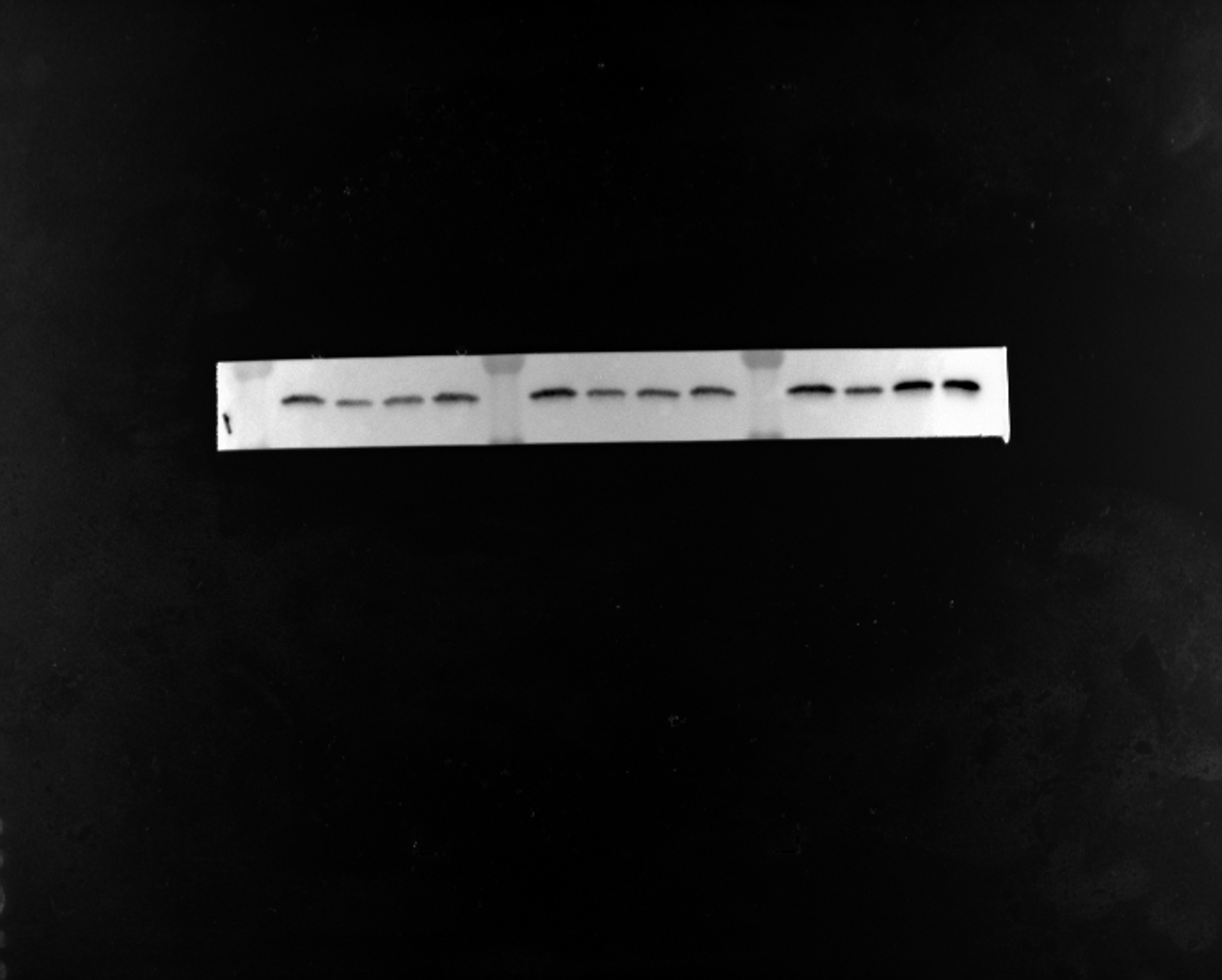

Supplement: Supplemental Information 2 [file peerj-12-16703-s002.zip › Figure 2-WB images/GPX4/GPX4.Tif]

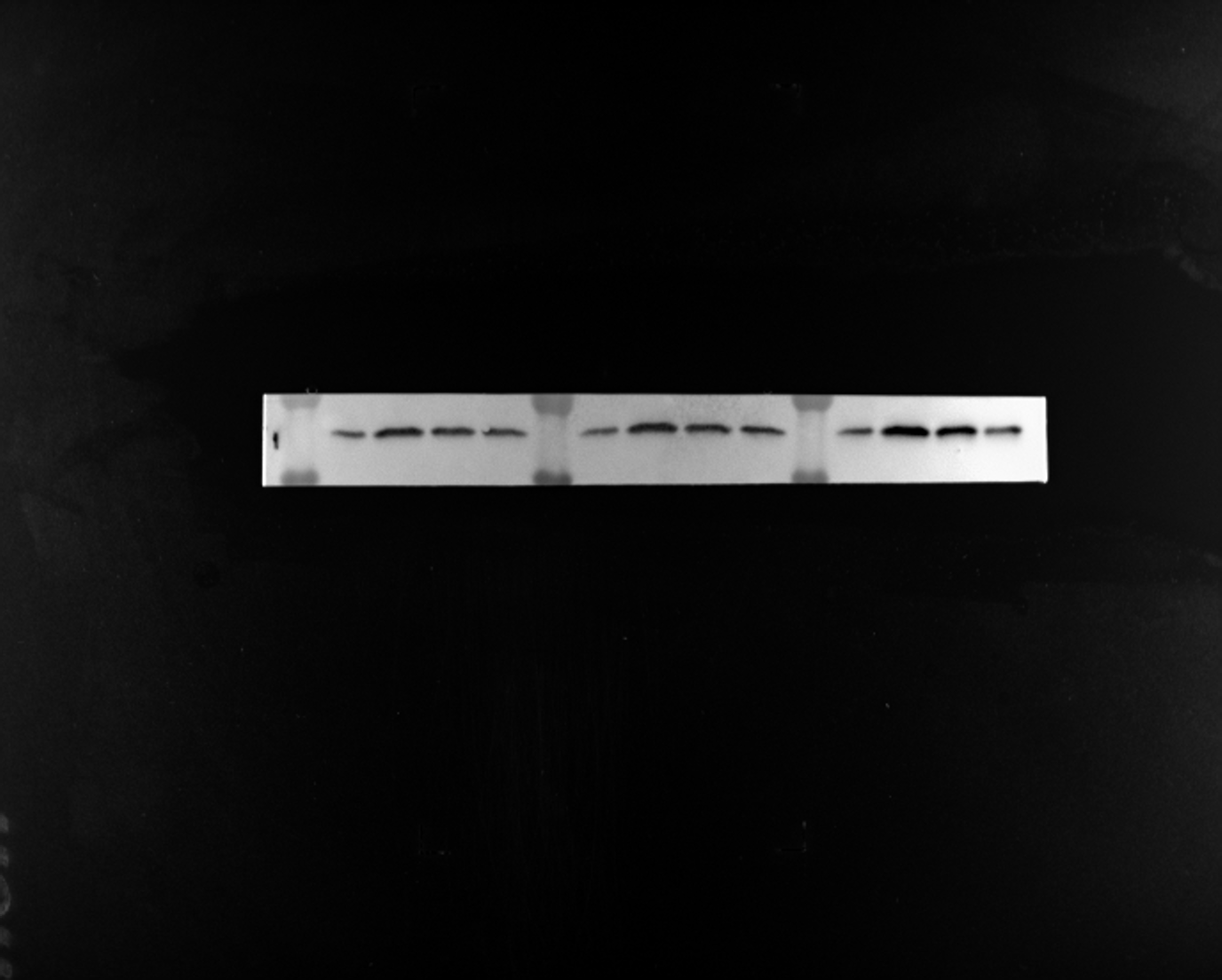

Supplement: Supplemental Information 3 [file peerj-12-16703-s003.zip › Figure 3-WB images/Collagen-I/collagen-I.Tif]

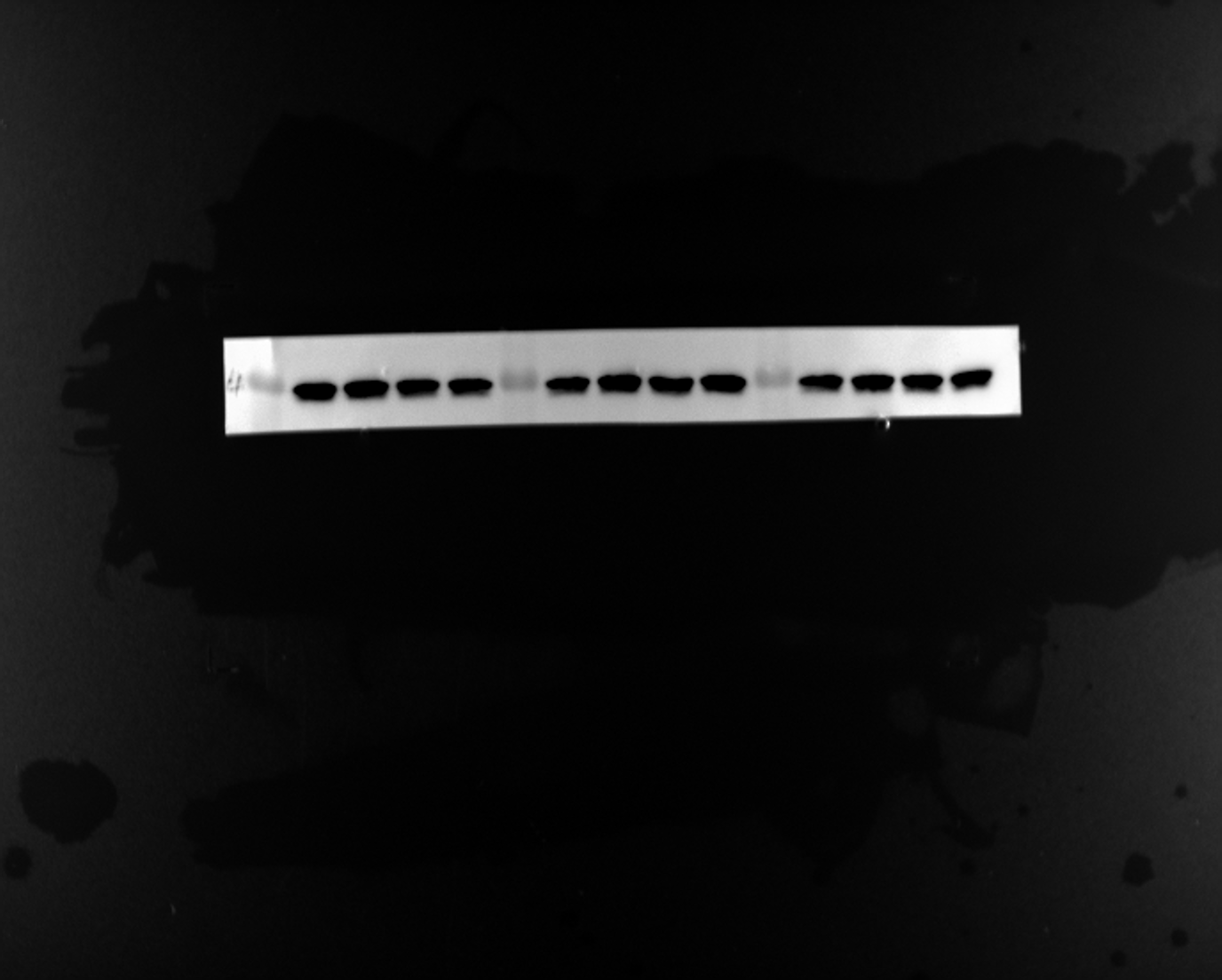

Supplement: Supplemental Information 3 [file peerj-12-16703-s003.zip › Figure 3-WB images/GAPDH-1/1-GAPDH.Tif]

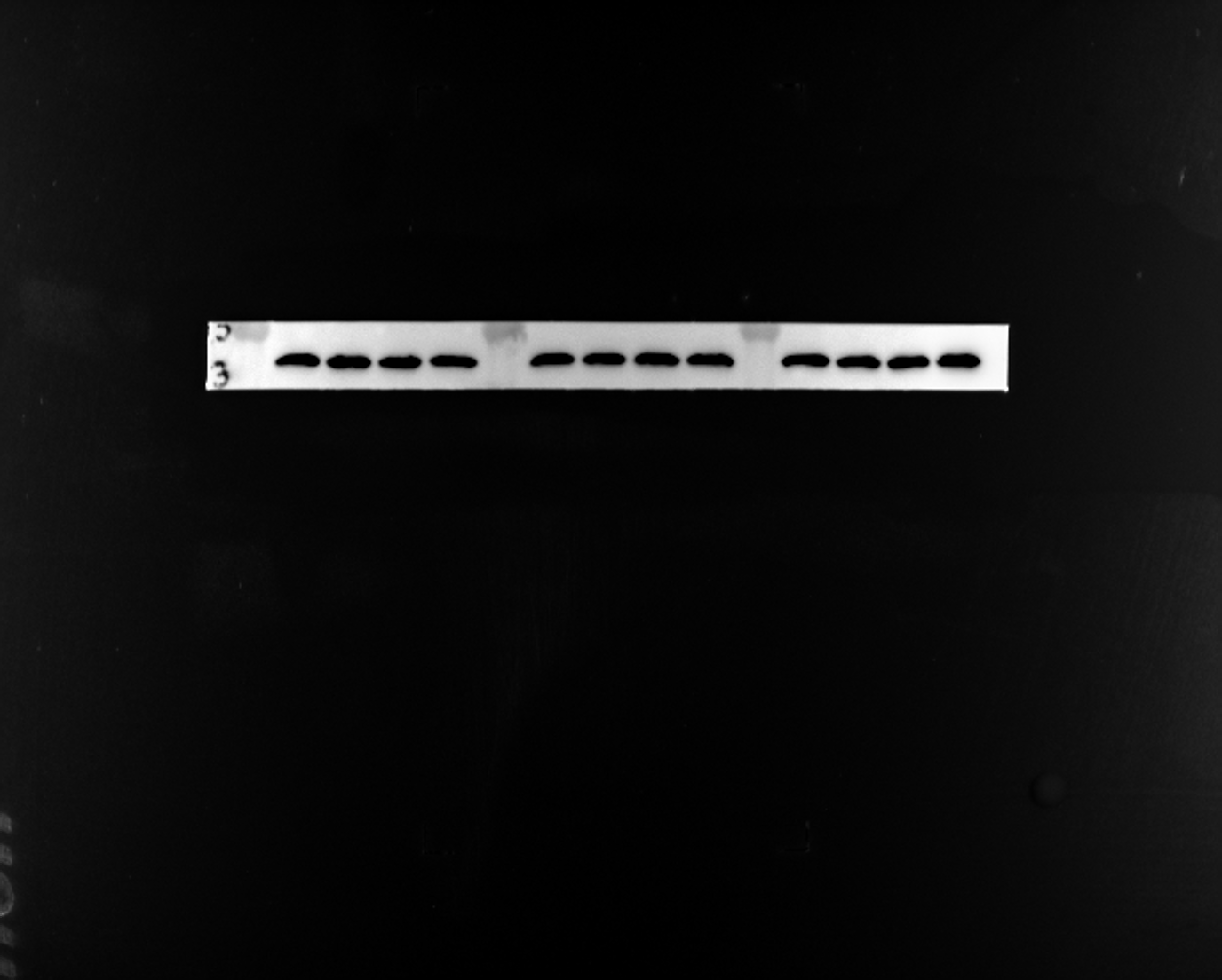

Supplement: Supplemental Information 3 [file peerj-12-16703-s003.zip › Figure 3-WB images/GAPDH-2/GAPDH.Tif]

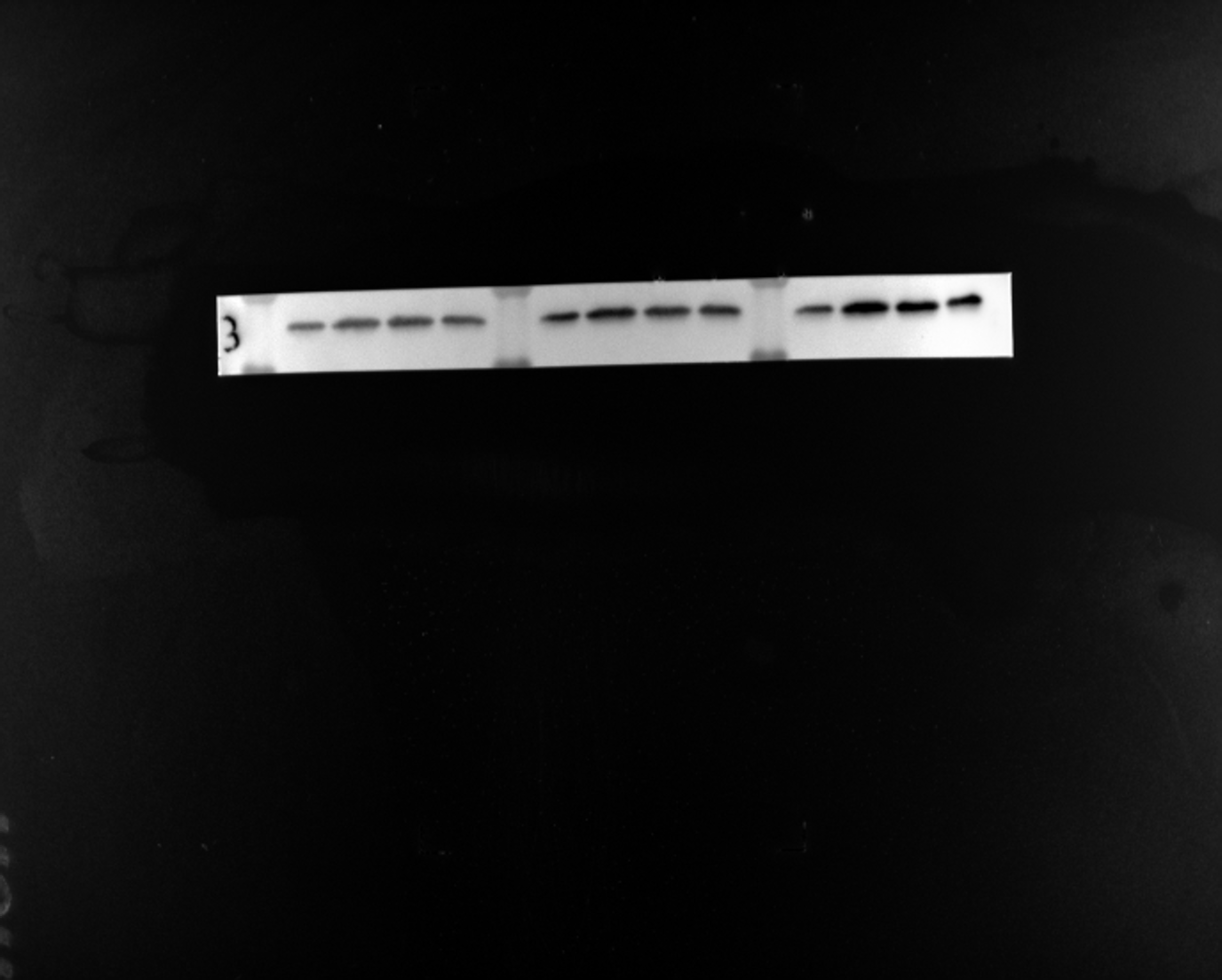

Supplement: Supplemental Information 3 [file peerj-12-16703-s003.zip › Figure 3-WB images/Keap1/Keap1.Tif]

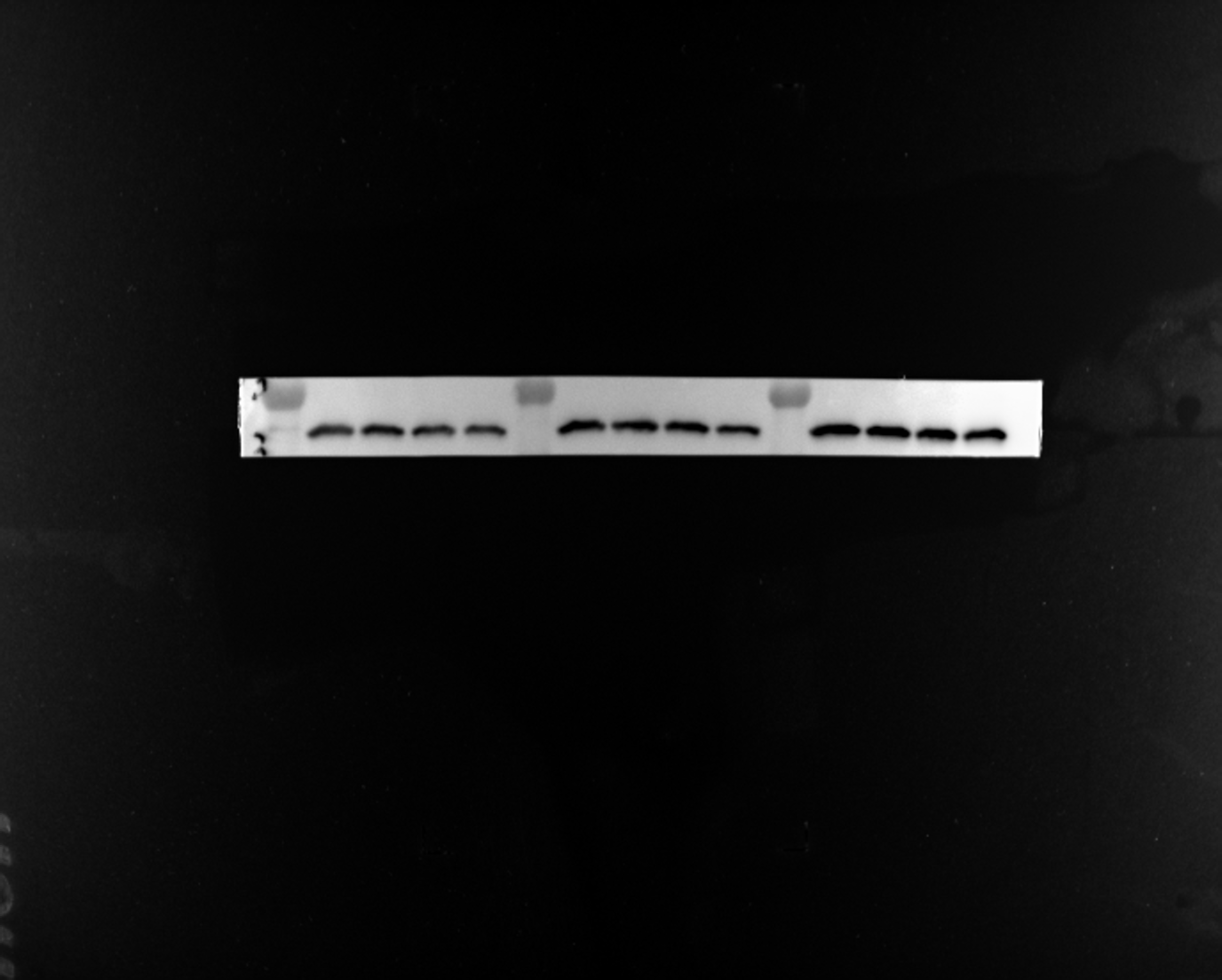

Supplement: Supplemental Information 3 [file peerj-12-16703-s003.zip › Figure 3-WB images/PCNA/PCNA.Tif]

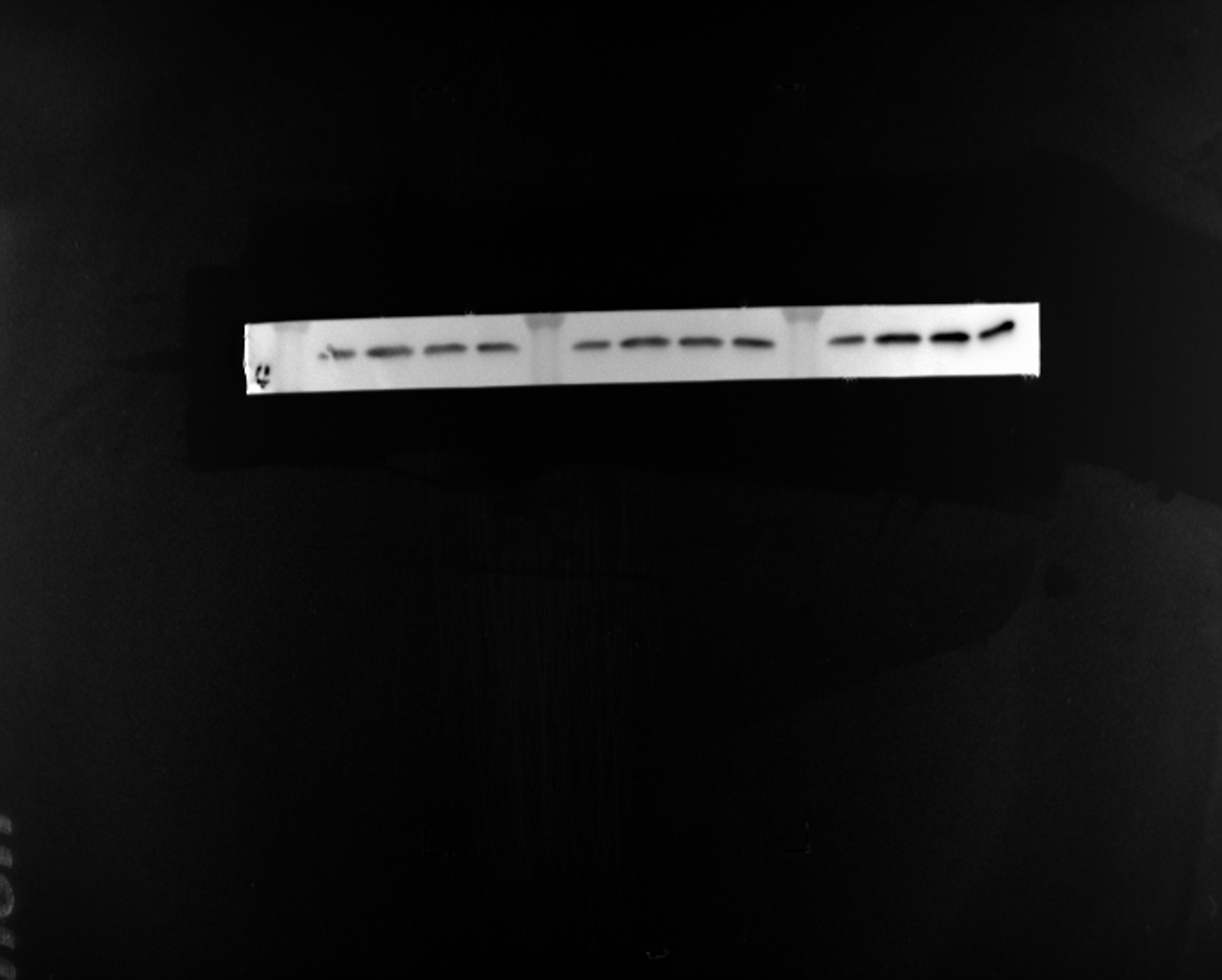

Supplement: Supplemental Information 3 [file peerj-12-16703-s003.zip › Figure 3-WB images/TGF-a┬1/TGF-a┬1.Tif]

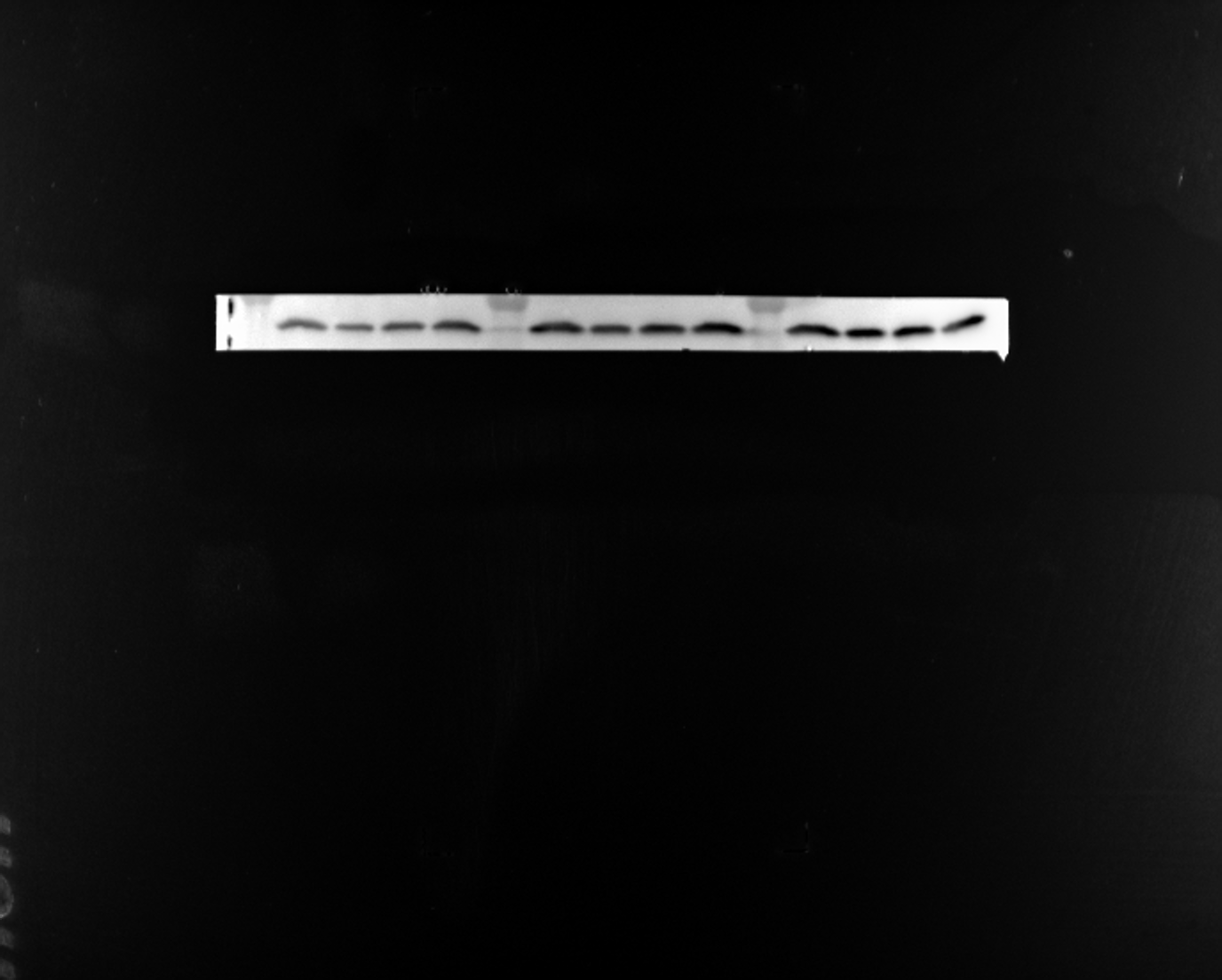

Supplement: Supplemental Information 3 [file peerj-12-16703-s003.zip › Figure 3-WB images/nulcear Nrf2/nuclear Nrf2.Tif]

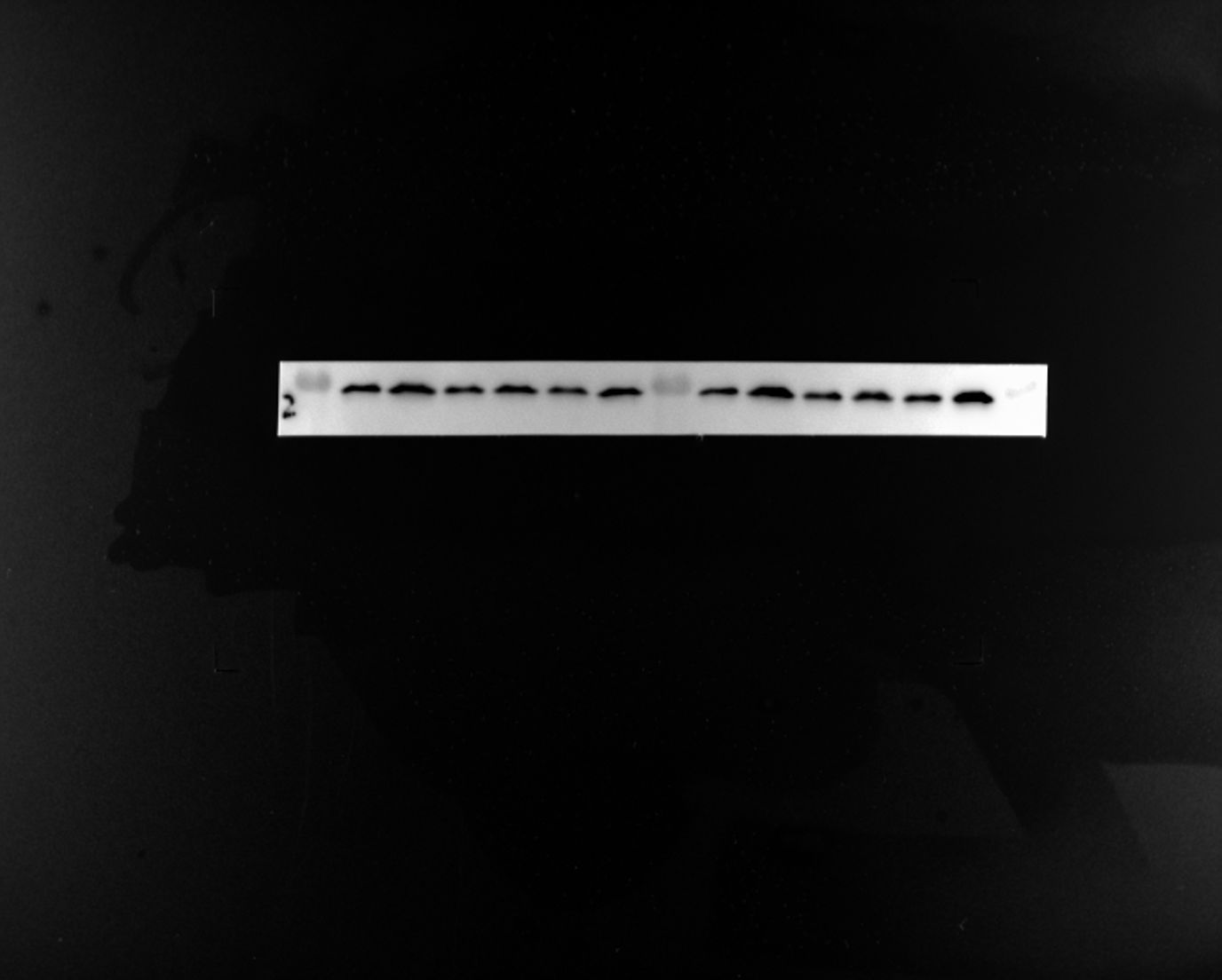

Supplement: Supplemental Information 4 [file peerj-12-16703-s004.zip › Figure 4-WB images/ACSL4/ACSL4-1 and 2.Tif]

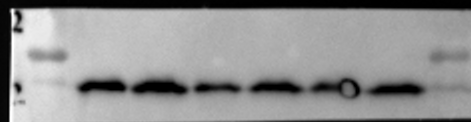

Supplement: Supplemental Information 4 [file peerj-12-16703-s004.zip › Figure 4-WB images/ACSL4/ACSL4-3.TIF]

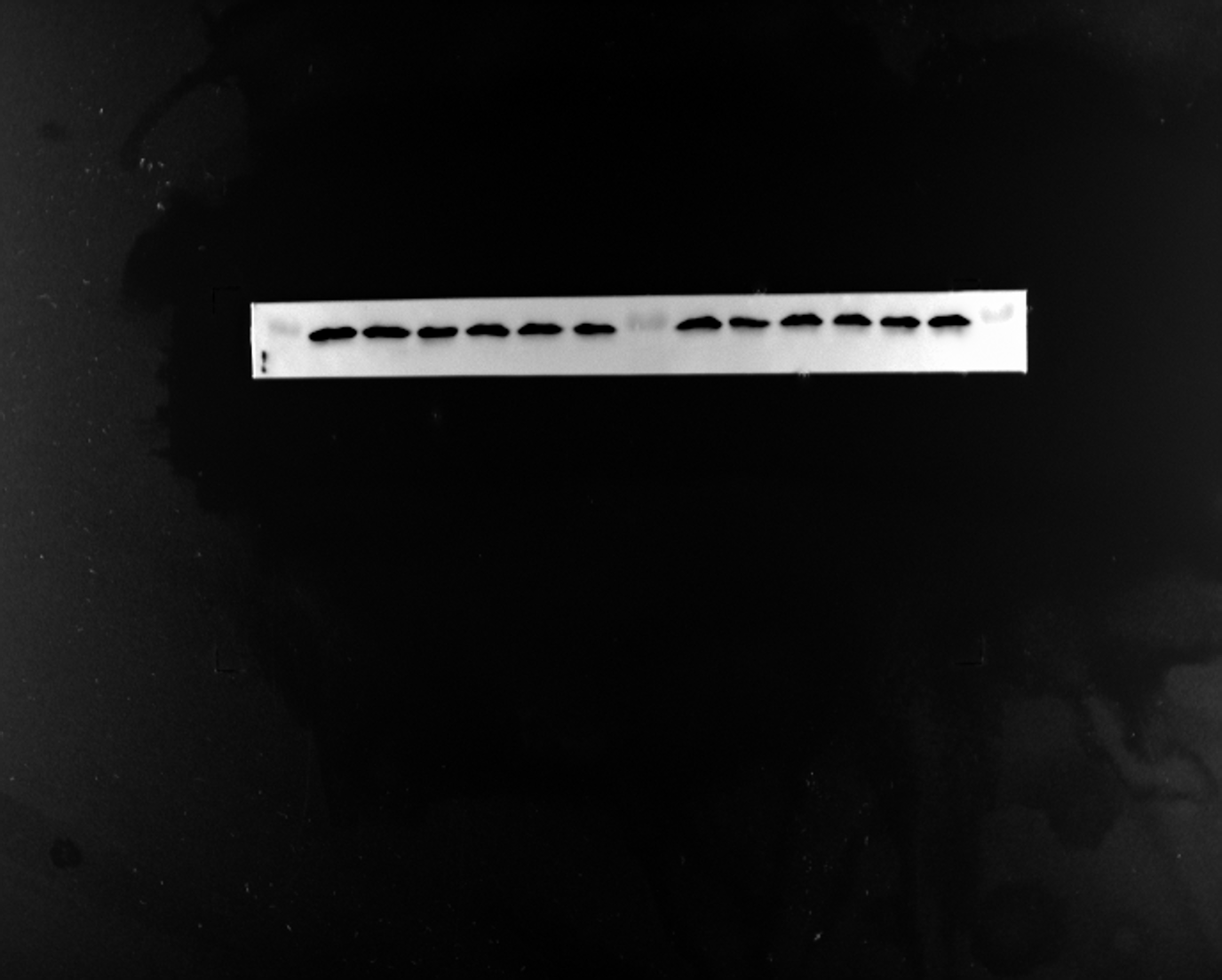

Supplement: Supplemental Information 4 [file peerj-12-16703-s004.zip › Figure 4-WB images/GAPDH/GAPDH-1 and 2.Tif]

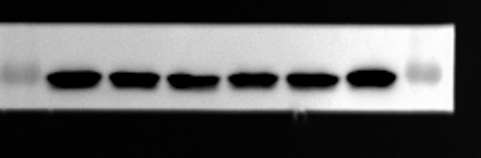

Supplement: Supplemental Information 4 [file peerj-12-16703-s004.zip › Figure 4-WB images/GAPDH/GAPDH-3.tif]

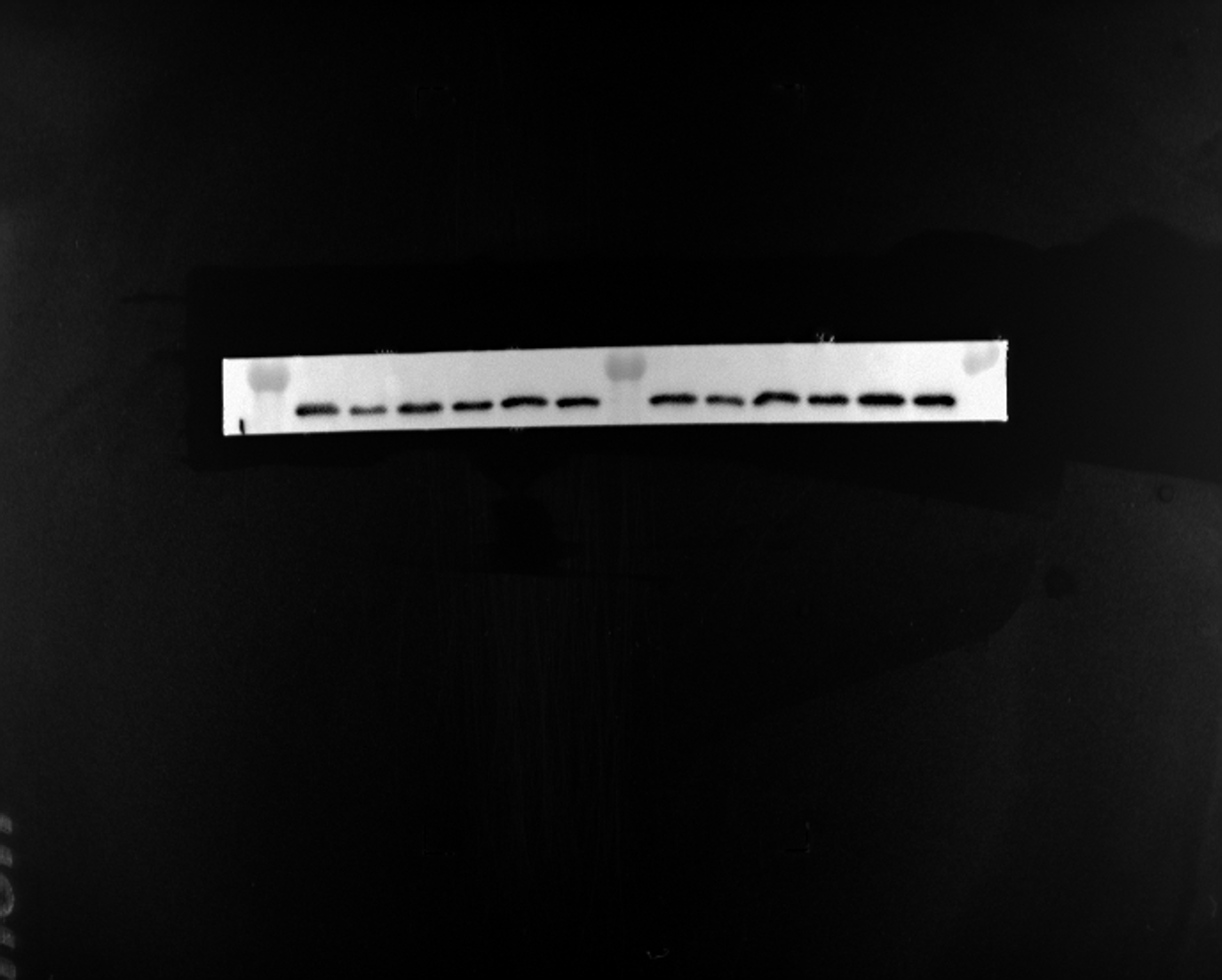

Supplement: Supplemental Information 4 [file peerj-12-16703-s004.zip › Figure 4-WB images/GPX4/GPX4-1 and 2.Tif]

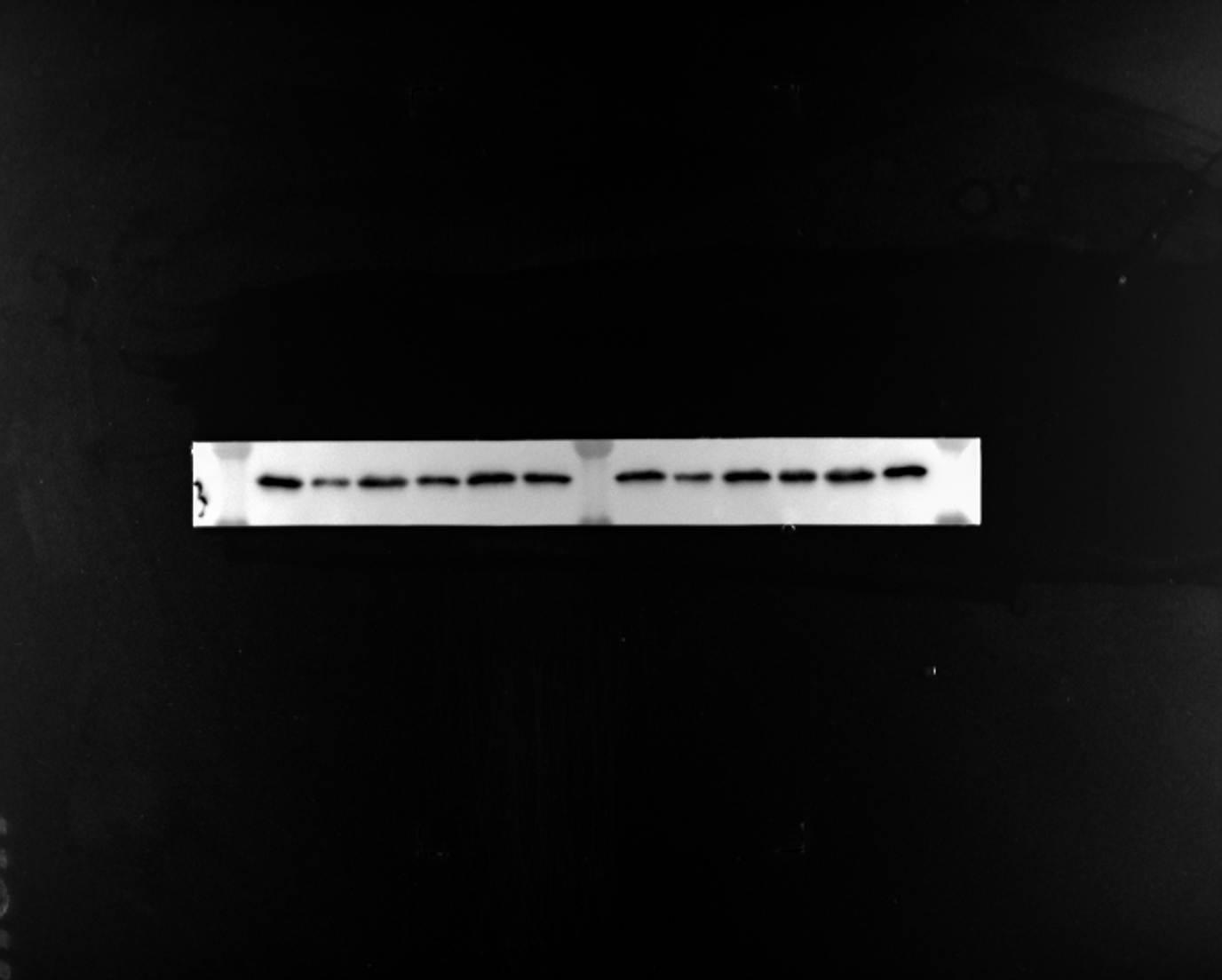

Supplement: Supplemental Information 4 [file peerj-12-16703-s004.zip › Figure 4-WB images/GPX4/GPX4-3.Tif]

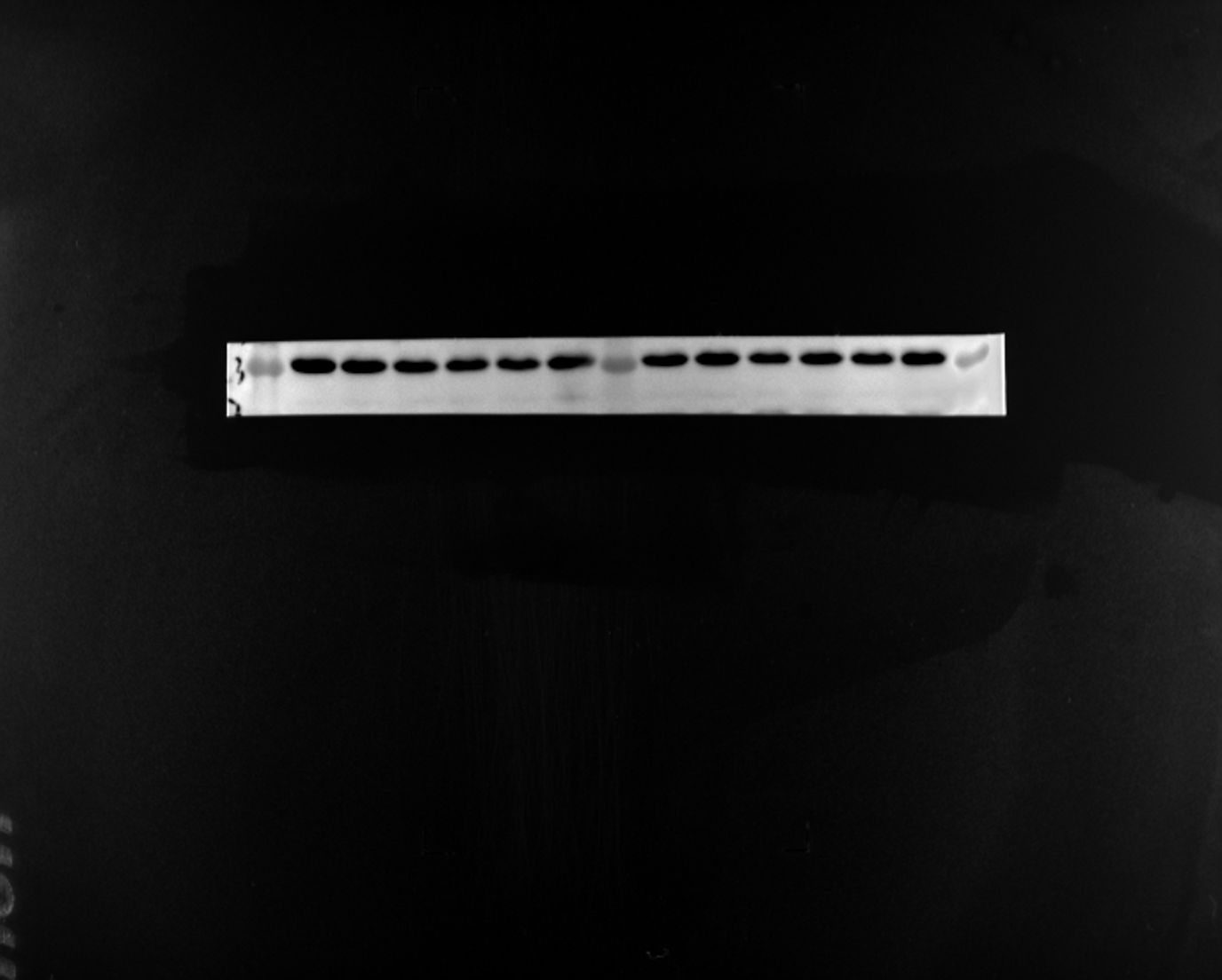

Supplement: Supplemental Information 5 [file peerj-12-16703-s005.zip › Figure 5-WB images/GAPDH/GAPDH-1 and 2.Tif]

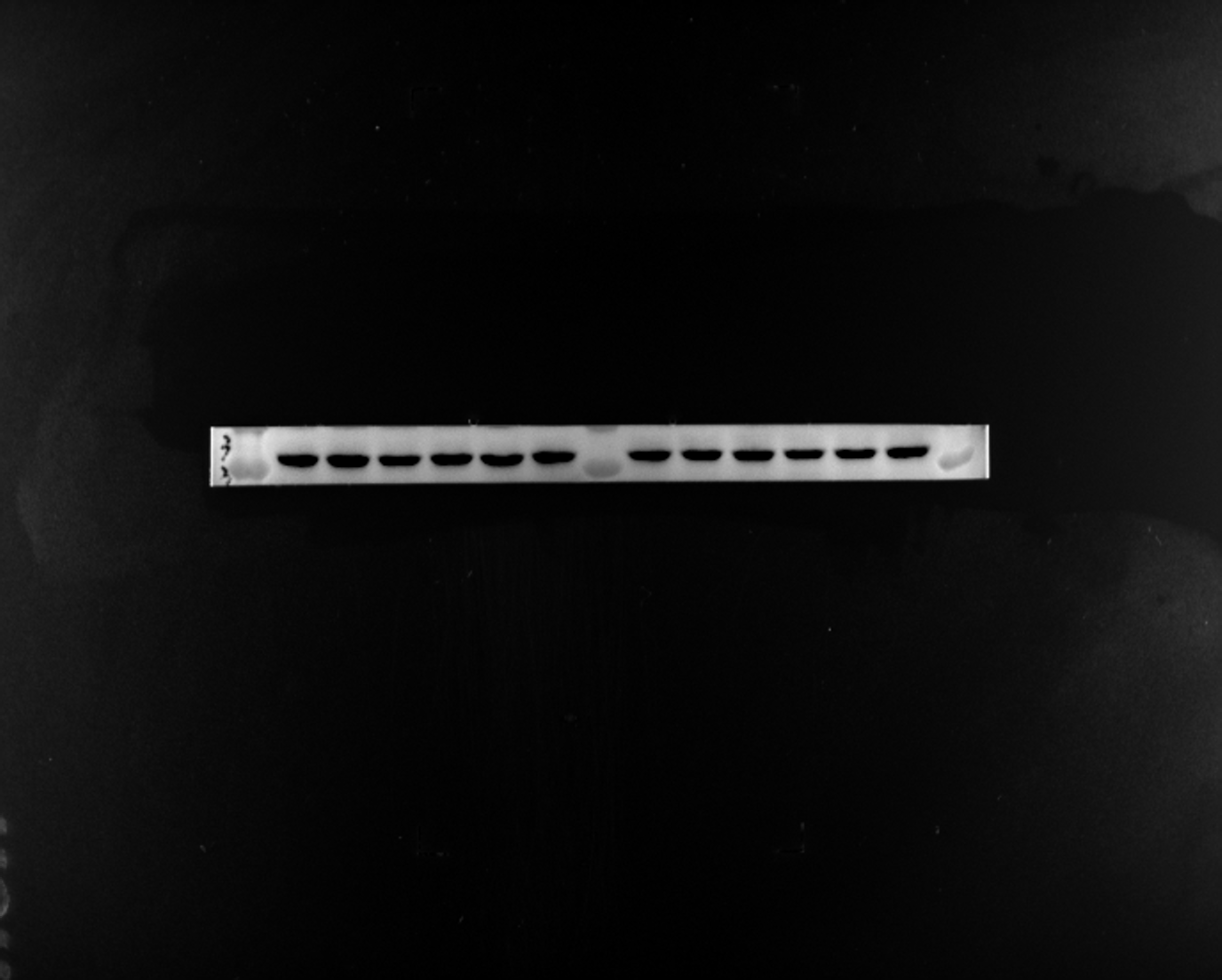

Supplement: Supplemental Information 5 [file peerj-12-16703-s005.zip › Figure 5-WB images/GAPDH/GAPDH-3.Tif]

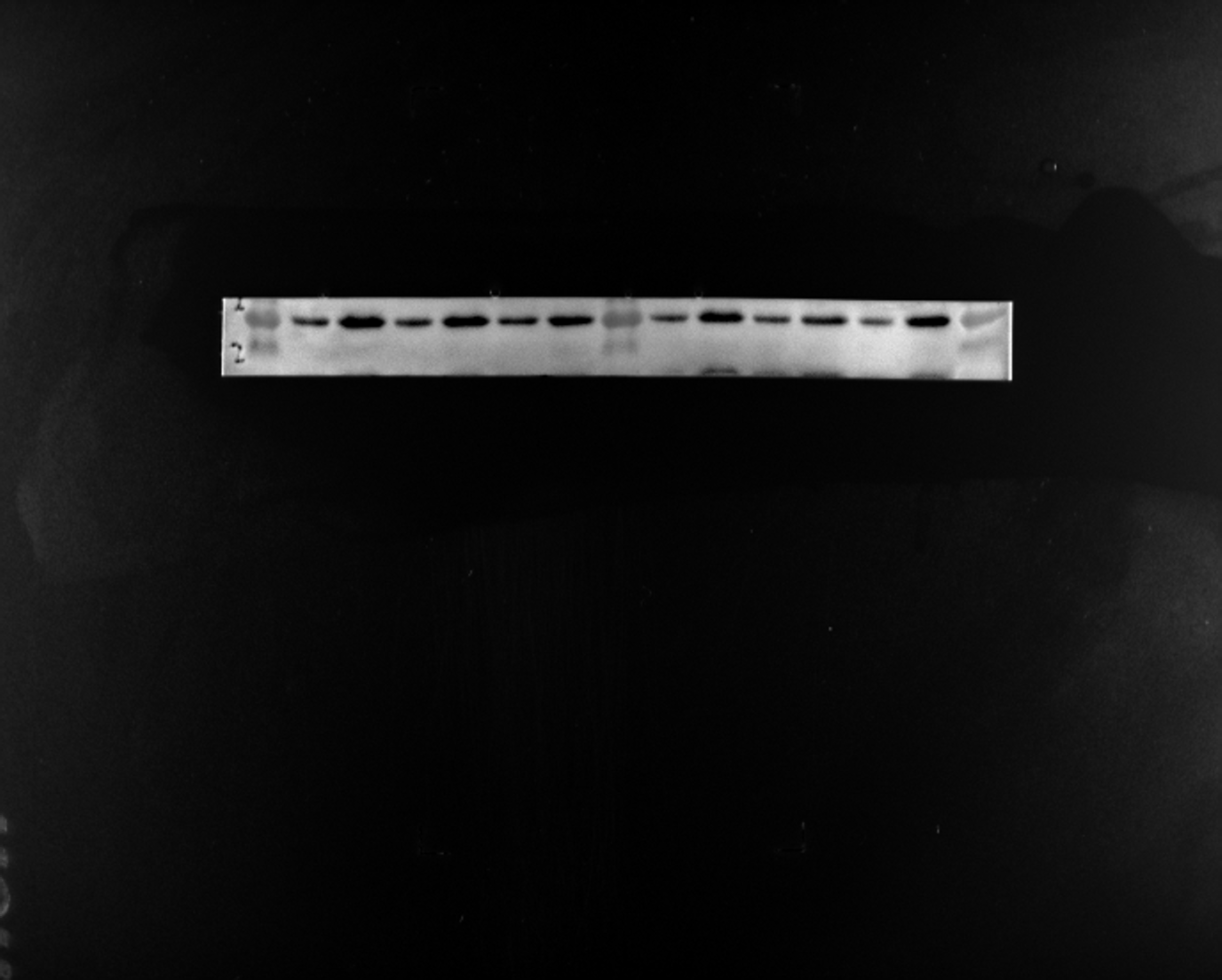

Supplement: Supplemental Information 5 [file peerj-12-16703-s005.zip › Figure 5-WB images/TGF-a┬1/TGF-a┬1-3.Tif]

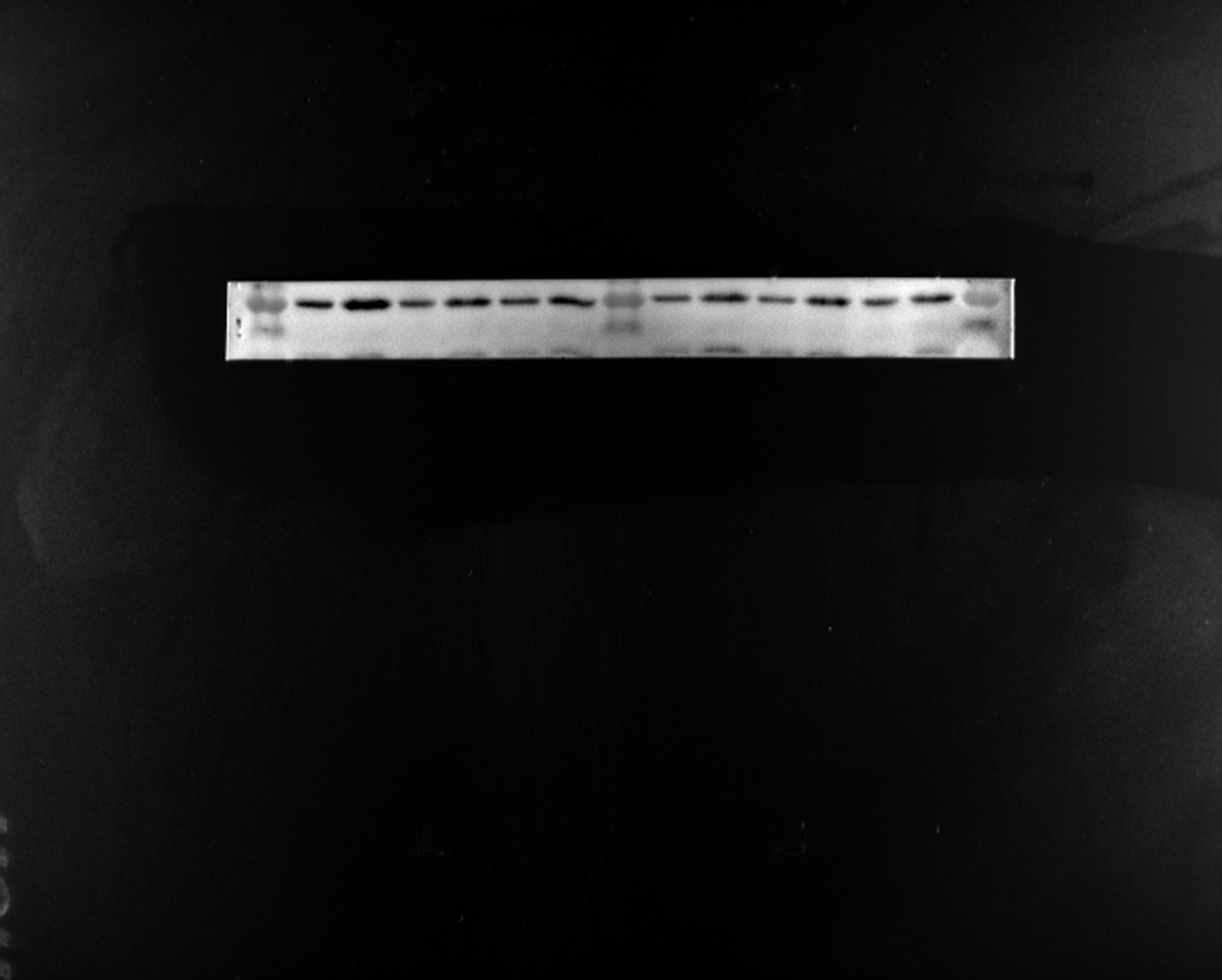

Supplement: Supplemental Information 5 [file peerj-12-16703-s005.zip › Figure 5-WB images/TGF-a┬1/TGF-a┬1.Tif]

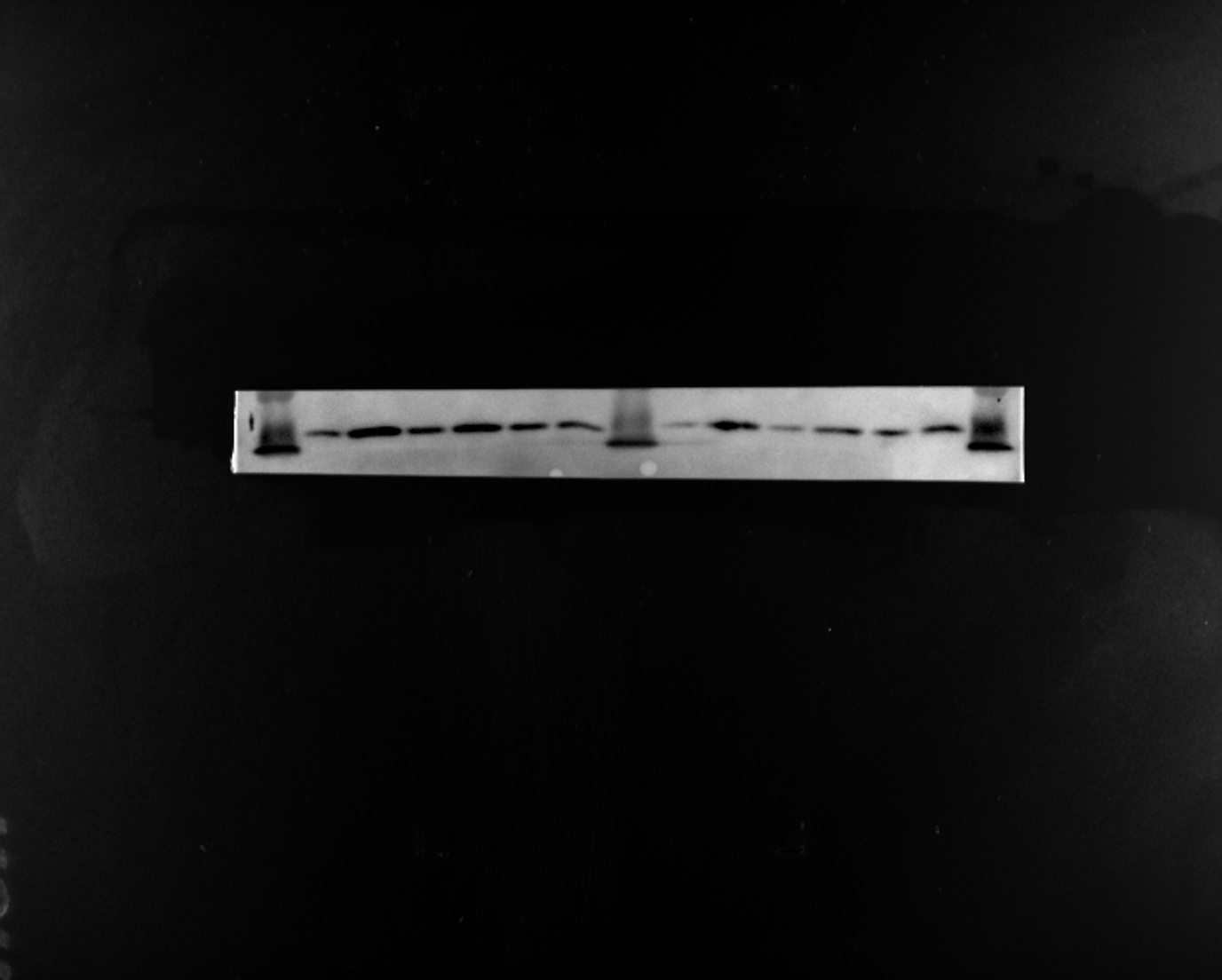

Supplement: Supplemental Information 6 [file peerj-12-16703-s006.zip › File5-Figure 6-WB images/Figure 6-WB images/ACSL4/ACSL4.Tif]

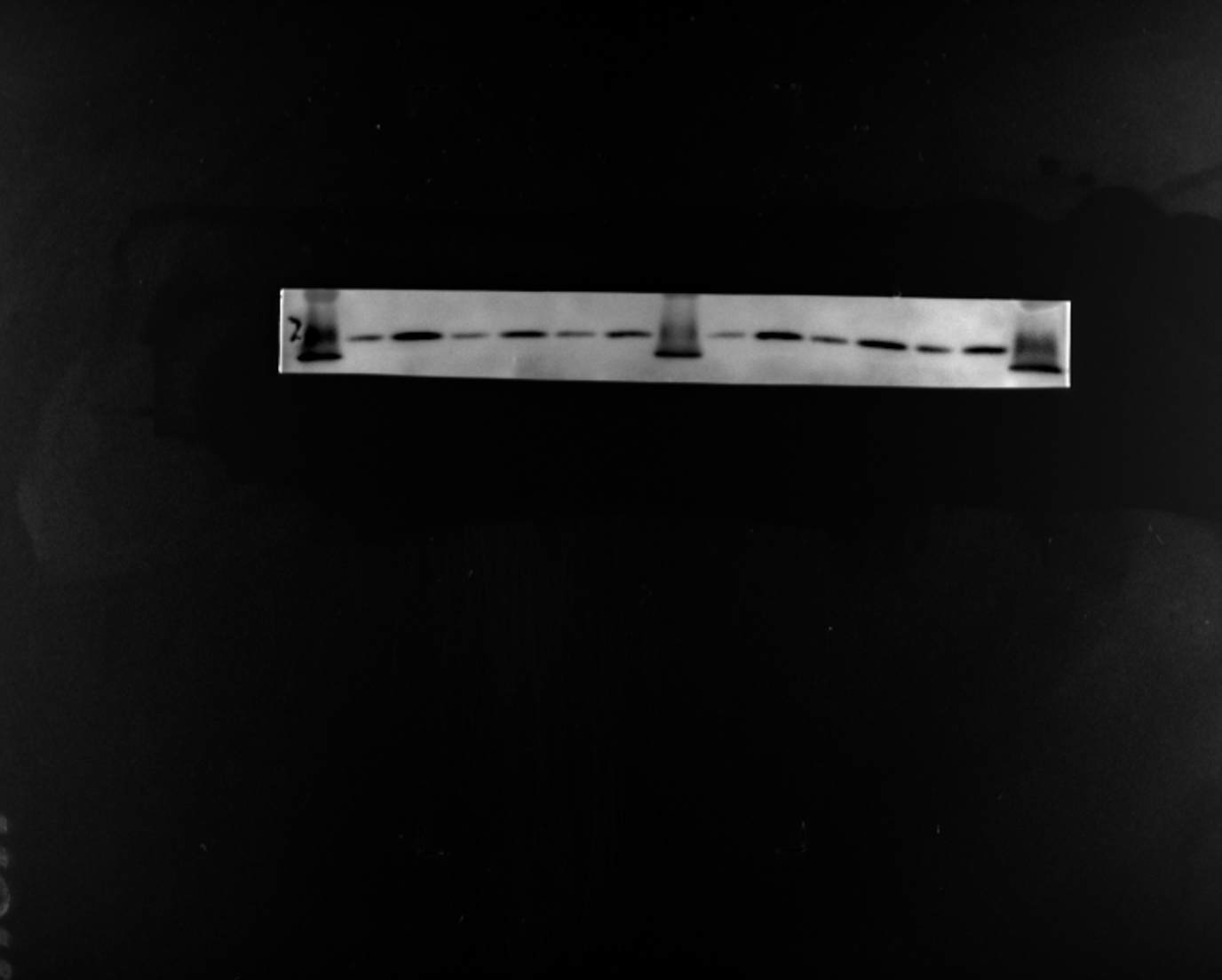

Supplement: Supplemental Information 6 [file peerj-12-16703-s006.zip › File5-Figure 6-WB images/Figure 6-WB images/ACSL4/ASCL4-1.Tif]

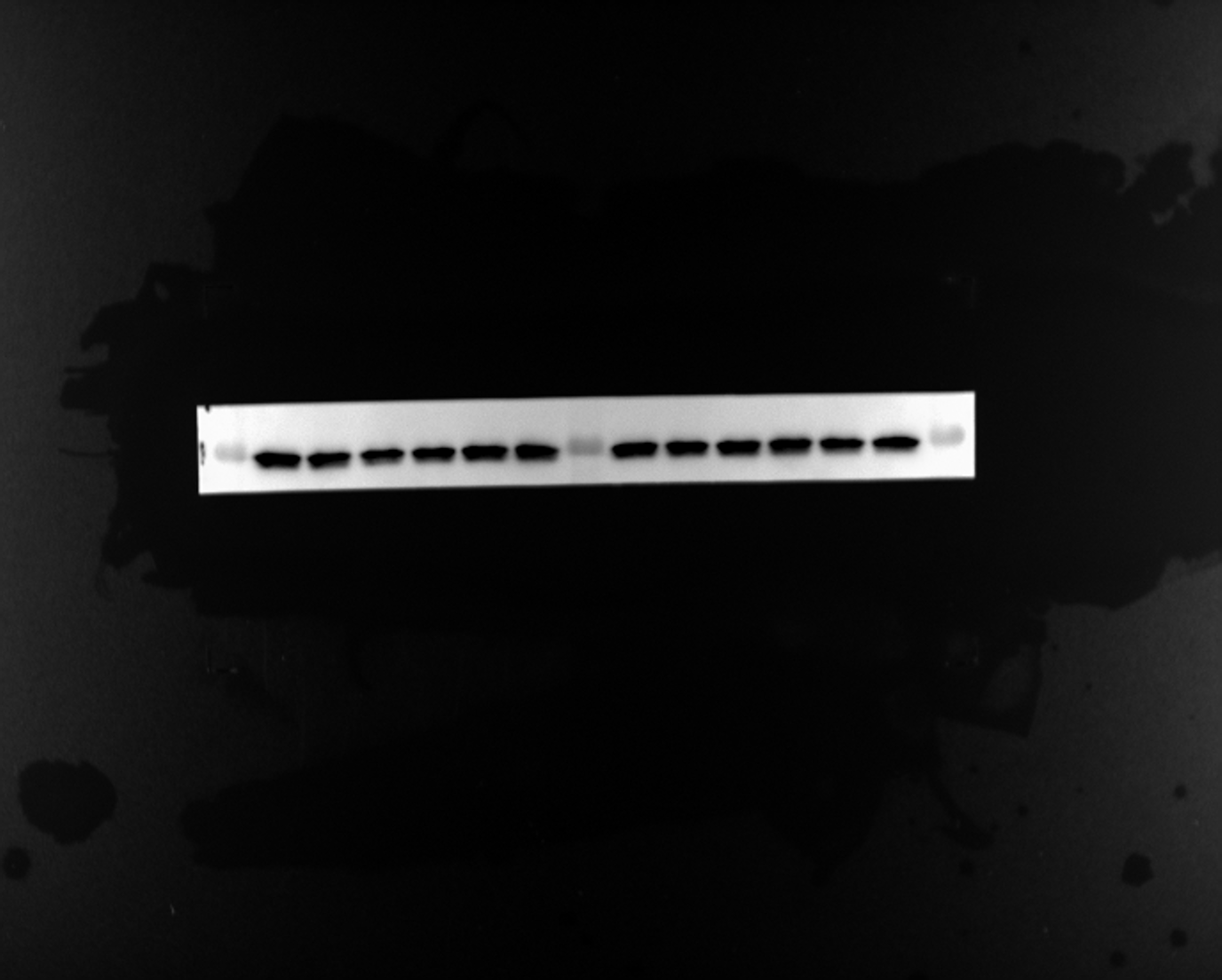

Supplement: Supplemental Information 6 [file peerj-12-16703-s006.zip › File5-Figure 6-WB images/Figure 6-WB images/GAPDH-1/GAPDH-1 and 2.Tif]

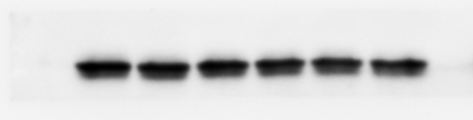

Supplement: Supplemental Information 6 [file peerj-12-16703-s006.zip › File5-Figure 6-WB images/Figure 6-WB images/GAPDH-1/GAPDH-3.tif]

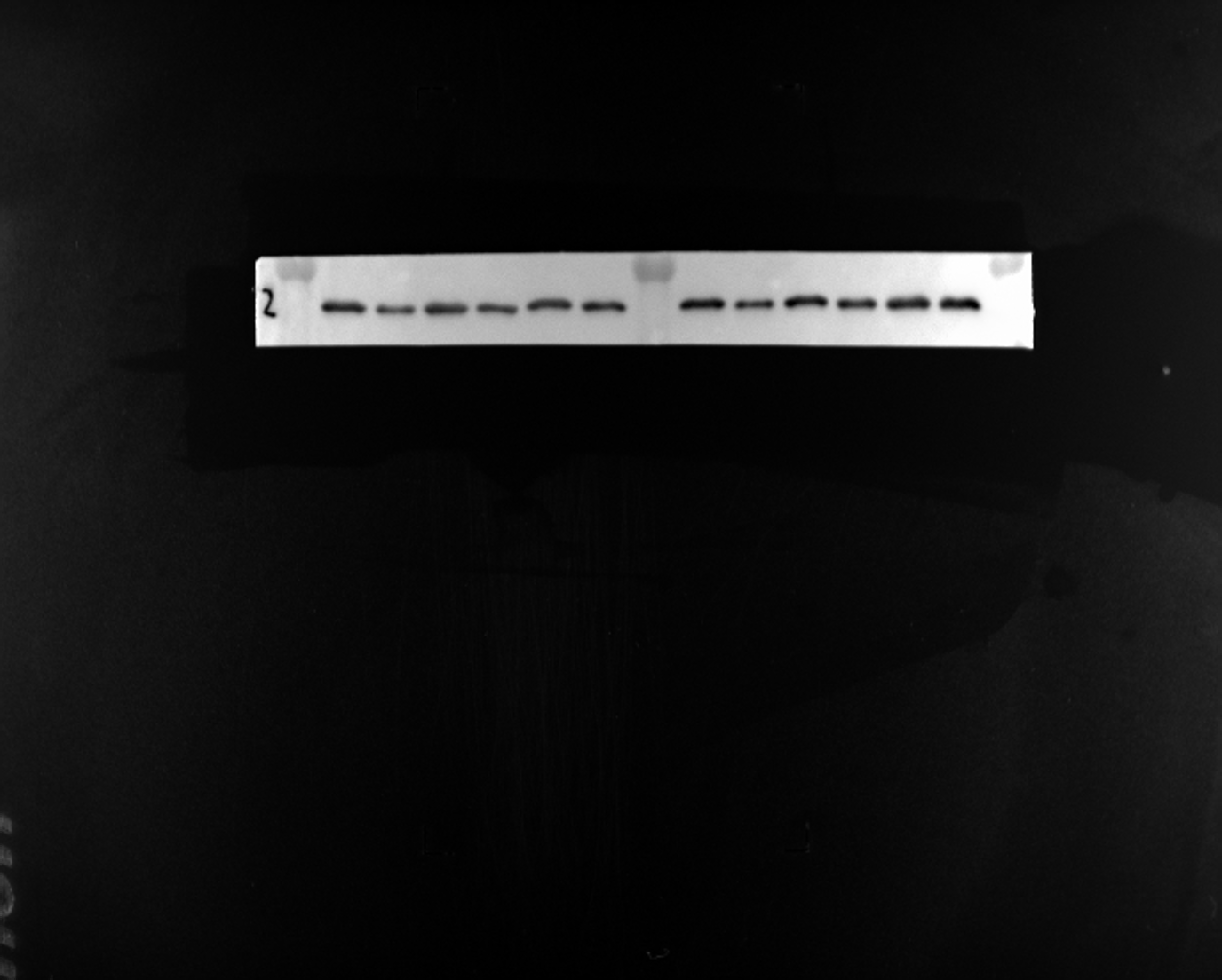

Supplement: Supplemental Information 6 [file peerj-12-16703-s006.zip › File5-Figure 6-WB images/Figure 6-WB images/GPX4/GPX4-1 and 2.Tif]

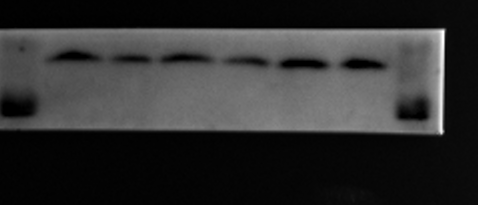

Supplement: Supplemental Information 6 [file peerj-12-16703-s006.zip › File5-Figure 6-WB images/Figure 6-WB images/GPX4/GPX4-3.tif]
